# Conserved Microsatellites in Ants Enable Population Genetic and Colony Pedigree Studies across a Wide Range of Species

**DOI:** 10.1371/journal.pone.0107334

**Published:** 2014-09-22

**Authors:** Ian A. Butler, Kimberly Siletti, Peter R. Oxley, Daniel J. C. Kronauer

**Affiliations:** Laboratory of Insect Social Evolution, The Rockefeller University, New York, New York, United States of America; Sheffield University, United States of America

## Abstract

Broadly applicable polymorphic genetic markers are essential tools for population genetics, and different types of markers have been developed for this purpose. Microsatellites have been employed as particularly polymorphic markers for over 20 years. However, PCR primers for microsatellite loci are often not useful outside the species for which they were designed. This implies that a new set of loci has to be identified and primers developed for every new study species. To overcome this constraint, we identified 45 conserved microsatellite loci based on the eight currently available ant genomes and designed primers for PCR amplification. Among these loci, we chose 24 for in-depth study in six species covering six different ant subfamilies. On average, 11.16 of these 24 loci were polymorphic and in Hardy-Weinberg equilibrium in any given species. The average number of alleles for these polymorphic loci within single populations of the different species was 4.59. This set of genetic markers will thus be useful for population genetic and colony pedigree studies across a wide range of ant species, supplementing the markers available for previously studied species and greatly facilitating the study of the many ant species lacking genetic markers. Our study shows that it is possible to develop microsatellite loci that are both conserved over a broad range of taxa, yet polymorphic within species. This should encourage researchers to develop similar tools for other large taxonomic groups.

## Introduction

Microsatellites, also called short tandem repeats (STRs) or simple sequence repeats (SSRs), are sequential repeats of 1 to 6 base pair motifs that have been used as genetic markers for more than 20 years [1], [2], [3]. Often found in noncoding regions, they are common in the genomes of eukaryotes [4]–[6]. An important feature of these sequences is their high degree of length polymorphism within populations of single species, which has been attributed to DNA polymerase slippage during replication [7], [8]. This can result in a large number of alleles per locus that differ from one another in the number of repeats, making them distinguishable by size alone. This high degree of polymorphism and the ease of genotyping make them particularly suitable for studies in population genetics and pedigree analyses [9], [10]. For example, microsatellites have been used to measure population differentiation and hybridization [11], [12], to investigate ploidy levels [13], [14], and to reconstruct parentage and pedigrees in wild and domestic populations [15], [16]. Microsatellites are comparatively cheap to genotype and can be used with low concentrations of DNA. Furthermore, they typically have more alleles per locus than single nucleotide polymorphisms (SNPs) and thus provide more information per locus [17]. Although they often have a high degree of polymorphism within species, some microsatellite loci can be conserved across species that diverged 100 million years ago or more [18]–[24].

More recently, next generation sequencing (NGS) techniques have risen in popularity, mainly because of the large number of marker loci they can generate at relatively low per locus cost. For example, restriction site-associated DNA (RAD) tags can generate thousands of markers and have proven instrumental for measuring gene flow between populations [25], as well as for reconstructing shallow phylogenies [26]. However, the data generated from these techniques can be complex and difficult to analyze. There are techniques to reduce the complexity of DNA libraries such as double digest RADseq (ddRAD) [27], 2b-RAD [28], or genotyping by sequencing (GBS) [29], but these still require expensive NGS platforms. On the other hand, for many studies a smaller number of markers is sufficient, and markers such as microsatellites can be more attractive.

Despite their utility, a significant impediment to the use of microsatellites is the cost and effort associated with identifying a set of loci and developing PCR primers. Although the same loci can sometimes be useful for studying closely related species, loci that are polymorphic in one species are often not informative in another, and primers quickly lose affinity as species become more divergent. This usually requires new microsatellite loci to be characterized for each studied species. Depending on the research question, studies typically require a set of five to ten or more independent microsatellite loci. Paying a commercial service to develop these markers can be costly, and developing markers independently can be labor intensive and time consuming.

Nevertheless, the utility of microsatellites in determining pedigree structures, relatedness and mating systems makes them particularly useful for social insect research because they can be used to address important questions related to inclusive fitness theory, including social organization (e.g. [30]), worker caste determination (e.g. [31]), and the evolution of supercolonies (e.g [32]). Of the social insects, ants are a particularly speciose and ecologically diverse group being intensively studied. Current estimates place the ant family Formicidae at 115 to 158 million years of age [33]–[35], and close to 13,000 species have been described, according to the Hymenoptera Name Server (v. 1.5, accessed 14 April 2014). Eight ant genomes are currently available representing most major ant clades, allowing highly conserved regions to be identified over most of the family. To help overcome the constraints of narrowly applicable primers and to make microsatellites broadly available as population genetic markers, we aimed to develop a set of microsatellite markers that would be conserved across a wide range of species, yet polymorphic within species.

## Results

**Figure 1:**
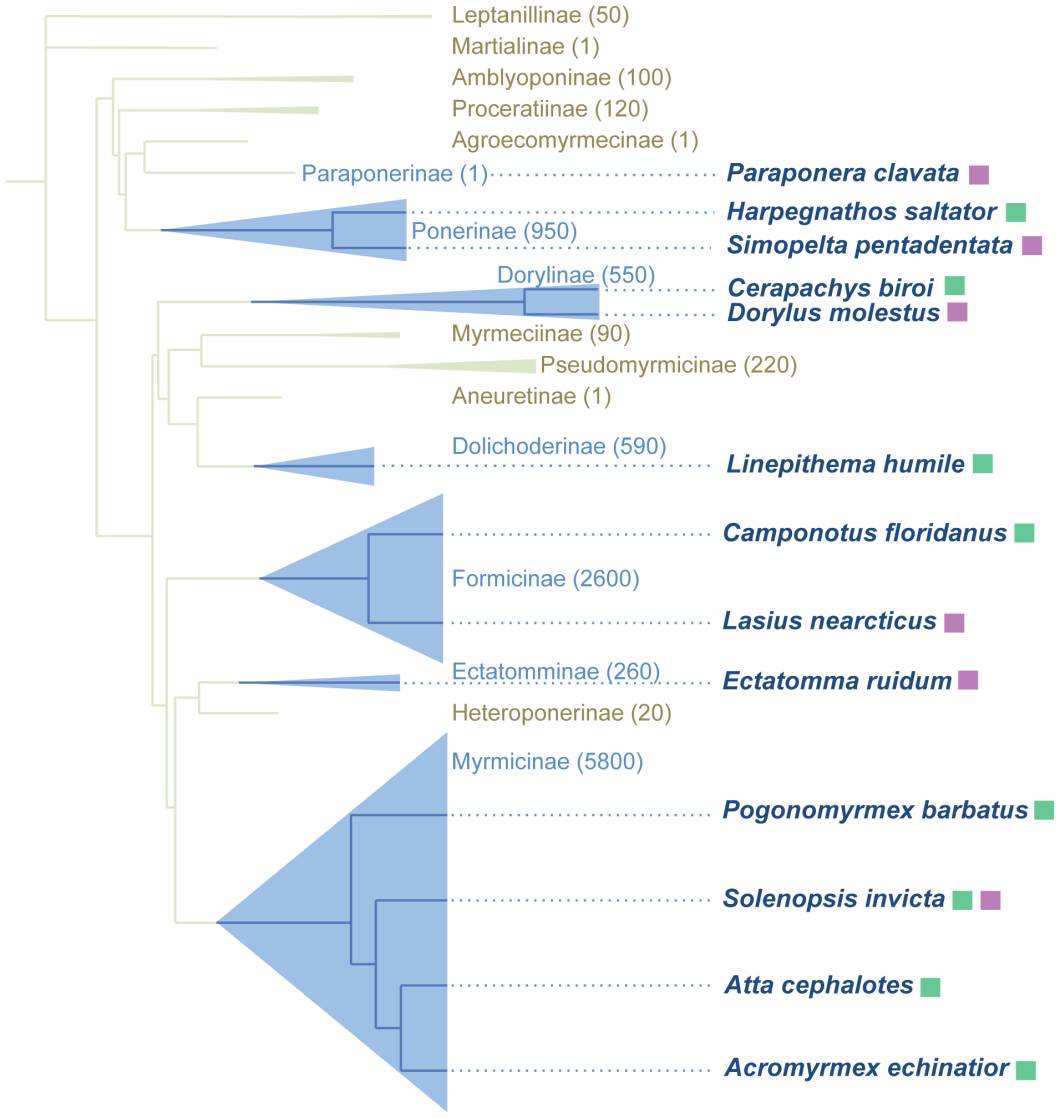
Phylogeny of the ants, showing the phylogenetic distribution of the species used in this study. The size of each triangle is proportional to the number of species in each group, and the approximate number of species is given in parentheses next to the group name. Boxes next to species names indicate whether that species' genome was used to design (green) or test (purple) the PCR primers. Figure adapted with permission from Libbrecht et al. 2013 [73].

To design a set of broadly applicable microsatellite primers we searched the eight currently available ant genomes for conserved microsatellite motifs with conserved flanking regions. The eight available ant genomes are from the red harvester ant *Pogonomyrmex barbatus* (subfamily Myrmicinae) [36], Jerdon's jumping ant *Harpegnathos saltator* (subfamily Ponerinae), the Florida carpenter ant *Camponotus floridanus* (subfamily Formicinae) [37], the leaf-cutting ants *Atta cephalotes* (subfamily Myrmicinae) [38] and *Acromyrmex echinatior* (subfamily Myrmicinae) [39], the Argentine ant *Linepithema humile* (subfamily Dolichoderinae) [40], the red imported fire ant *Solenopsis invicta* (subfamily Myrmicinae) [41], and the clonal raider ant *Cerapachys biroi* (subfamily Dorylinae) [42]. The available genomes represent five of the 21 recognized extant ant subfamilies, allowing us to select primer sequences that are conserved in a wide range of species across the ants (Figure 1). We identified 176 potential microsatellite loci with conserved flanking regions across all eight genomes, and among those selected 45 that had a repeat motif in most or all of the available genomes (Table S1 in File S1). To demonstrate their usefulness in species other than those with available genomes, we tested these primers for amplification in six species from six different subfamilies, only one of which was also used for primer design (*Solenopsis invicta*, subfamily Myrmicinae) (Figure 1). The other five species in which the markers were tested were the bullet ant *Paraponera clavata* (subfamily Paraponerinae), the army ants *Simopelta pentadentata* (subfamily Ponerinae) and *Dorylus molestus* (subfamily Dorylinae), *Lasius nearcticus* (subfamily Formicinae), and *Ectatomma ruidum* (subfamily Ectatomminae). The success of PCR amplification varied by locus and species (Tables 1 & 2).

**Table 1:** PCR amplification success across six ant species for the 21 microsatellite loci that were only tested with unlabeled PCR primers.

Locus	Primer sequence (5'-3')	*P. clavata*	*S. pentadentata*	*D. molestus*	*L. nearcticus*	*E. ruidum*	*S. invicta*
Ant21	F-TTCTCGGGAGCAACCGTGGTR-CCATCACGCACTCCACCTCG	Yes	Yes	Yes	Yes	Yes	Yes
Ant608	F-AGCGGATCTAGTGGTCTTGGR-ATGGAGGGGAGTAAGAGCGA	Yes	Yes	Yes	Yes	Yes	Yes
Ant1049	F-GAGGATGCGGTGGTGGCGGAR-CTGCGCCGCTCCGTGTGTAT	Yes	Yes	No	Yes	Yes	Yes
Ant1052	F-GCGACCTTCGTGCACGGTATCR-CTTTTAGTCAGACGCACGCG	Yes	Yes	No	Yes	Yes	Yes
Ant1387	F-ATAGGTGCCACATACGCGTGR-CACAGCCGACTCCCCTCTCC	Yes	Yes	Yes	Yes	Yes	Yes
Ant1732	F-ATGATACGCATGCGAGTGCCR-GCCAGCTCCTCCGAGCCTAT	Yes	No	No	Yes	Yes	Yes
Ant2409	F-ATCAGCGTCACGATCGAGTTR-CGTGATTCTTCTGACGCGAC	Yes	Yes	No	Yes	Yes	Yes
Ant3362	F-CCCCAAAACCTACCTCGTCCR-GTCTACAAGCTCGCGATGGA	Yes	Yes	No	Yes	Yes	Yes
Ant3395	F-CCRACGGGCGTCGGCAGTCCR-CCGGCACTTGGTACACGGTA	Yes	Yes	Yes	Yes	Yes	Yes
Ant3411	F-GCGGCAGCAGCGATCACCCCR-TGCAGCAGGACCGCCGTRGT	Yes	Yes	Yes	Yes	Yes	Yes
Ant3452	F-TGTGGAGTGCGGCARTGGGAR-ATCGACGACAAATCGTGGGC	No	Yes	No	Yes	Yes	Yes
Ant3505	F-TTACCGGACAATCGTGGTGGR-TGAGCACAGCACGACATTCT	Yes	No	Yes	Yes	Yes	Yes
Ant3541	F-TGCAACAAGTGTCCTGAGGTR-TCACATGTTCCGGCGYGCAT	No	No	No	No	No	No
Ant4709	F-ACGGGGTAAAGGGTTAGGGAR-AGCGATGGGAGATTGGAGAG	No	No	MP	Yes	Yes	Yes
Ant5033	F-TTCCCCTCTCCCTGACCACCR-TAAGACAAGGAACGTCCGCG	Yes	No	Yes	Yes	No	Yes
Ant7204	F-GCCCAATCCTCTGCATTCCTR-CCCGCGAAAAGTCCATTTCGC	Yes	Yes	MP	Yes	Yes	Yes
Ant8544	F-GGGGTGCGTGCCAGTCTCGTR-CAATGCGATCTAGGTCACCA	Yes	Yes	Yes	MP	No	Yes
Ant9564	F-TTAGAGGCGCCAGSCTGCTR-AGCGAGCAACTTCGATGACT	Yes	Yes	No	Yes	Yes	Yes
Ant10290	F-CGTTTTCAAATTAACGTTTTTGCCR-ACGCGCGCTTCCGCGCTCGGG	No	No	No	No	No	No
Ant10427	F-AATCAGCTTAGCCGCGCTAAR-ATCCACCGCATCTGGGATTC	Yes	Yes	No	Yes	No	Yes
Ant11610	F-GGATAYTGGGGCGGCGTCAAR-GCCGAAAGTGTGGATACCTC	No	No	No	No	No	No

See Table 2 for details on the remaining 24 loci that were also tested using labeled PCR primers. “Yes” indicates clear amplification of a single product. “No” indicates no amplification of any product. “MP” indicates that there were multiple products from which the desired product could not be determined.

**Table 2:** Characteristics of 24 microsatellite loci tested in six different ant species.

		*Paraponera clavata*						*Simopelta pentadentata*						*Dorylus molestus*						*Lasius nearcticus*						*Ectatomma ruidum*						*Solenopsis invicta*					
Locus	Primer sequence (5'-3')	*n*	*A*	Size range (bp)	H_O_	H_E_	Deviates from HWE	*n*	*A*	Size range (bp)	H_O_	H_E_	Deviates from HWE	*n*	*A*	Size range (bp)	H_O_	H_E_	Deviates from HWE	*n*	*A*	Size range (bp)	H_O_	H_E_	Deviates from HWE	*n*	*A*	Size range (bp)	H_O_	H_E_	Deviates from HWE	*n*	*A*	Size range (bp)	H_O_	H_E_	Deviates from HWE
Ant20	F-AGGTCCTAGCAGGTAACATTGR-CCTCGGTCGATCGAGCGAGC	10	1	137	0	0	no	10	3	171–177	0.3	0.54	no	10	1	150	0	0	no	10	1	74	0	0	no	10	1	140	0	0	no	10	1	153	0	0	no
Ant575	F-TCAGGTTCGACACATGTGCCR-TCAAGATCGTTTGTCAGGCTGA	10	4	370–379	0.9	0.63	no	10	11	334–375	0.4	0.96	yes	10	2	230–250	0.2	0.19	no	10	4	209–234	0.3	0.37	no	10	1	248	0	0	no	10	3	218–239	0.7	0.63	no
Ant859	F-TACGCGGAGAAACGTCTGGTR-GTGATCTAAACTTCGATGAAC	10	5	184–206	0.7	0.77	no	10	3	180–184	0.3	0.54	no	10	2	197–199	0.2	0.19	no	9	11	175–204	1	0.94	no	10	1	158	0	0	no	10	1	191	0	0	no
Ant1343	F-TCGGTCCCGTGCCTTCGATTR-GRGGGCGCGTCAAATTTGCT	10	4	229–235	0.6	0.53	no	10	1	186	0	0	no	10	4	263–269	0.6	0.76	no	10	3	206–211	0.4	0.58	no	10	1	221	0	0	no	10	4	252–272	0.9	0.71	no
Ant1368	F-ACTACCCCAATGACGACACGR-CTATGCAGGTGCGGGTGTAT	10	1	251	0	0	no	7	6	266–313	0.14	0.93	yes	10	8	299–322	0.9	0.85	no	10	5	278–309	0.6	0.62	no	10	1	269	0	0	no	10	1	280	0	0	no
Ant2341	F-RAACAGCAGCTGTCCGGAGGR-GTCGCTGATCGCCACGTTCC	no amplification						10	5	345–359	0.7	0.76	no	10	4	256–267	0.2	0.55	no	10	2	212–215	0.4	0.51	no	10	1	184	0	0	no	10	2	245–251	0.3	0.27	no
Ant2794	F-TGGTGTGCGTGTTTGCRAGGR-GACTGCCAACCTACGGACTC	10	3	241–251	0.5	0.42	no	9	9	280–336	0.67	0.90	no	10	5	246–270	0.4	0.77	no	10	10	240–268	1	0.89	no	10	1	218	0	0	no	9	1	258	0	0	no
Ant2936	F-GGGGGATCCGGTAATCCTCTR-TCGCCCTGCAGTTAATGTGT	no amplification						no amplification						10	7	314–336	0.3	0.92	yes	10	9	352–390	0.4	0.9	no	no amplification						10	5	349–365	0.1	0.81	yes
Ant3648	F-CTCCTGGTCCTGGATCTCCAR-TAACACCATGCCCTCTGTCG	9	1	337	0	0	no	10	10	368–410	0.5	0.94	yes	10	7	376–421	0.5	0.83	no	10	3	332–343	0.6	0.57	no	10	4	393–401	0.3	0.67	no	10	1	337	0	0	no
Ant3653	F-AGCAGAGACCAATCAACGGAR-GGCAATTATCGGACCGGGTT	10	1	273	0	0	no	10	9	238–254	0.8	0.85	no	10	3	255–259	0.6	0.62	no	10	9	261–319	0.4	0.9	yes	10	4	357–363	0.4	0.74	no	10	2	254–256	0.4	0.33	no
Ant3993	F-TGATCCGCTCTTAAAATTTAGATGGAR-ACTTTCCGCRGCATTAAACATTTTCTT	8	7	368–387	0.88	0.88	no	10	5	368–379	0.2	0.81	yes	10	2	311–317	0.5	0.48	no	10	7	379–419	0.7	0.77	no	8	1	454	0	0	no	10	3	375–363	0.7	0.47	no
Ant4155	F-AGAATCTCTTGAGCCCGTCGR-GGCGATACACTTCACCTGAGAC	10	1	162	0	0	no	8	3	206–211	0.38	0.64	no	10	4	176–195	0.8	0.61	no	10	1	170	0	0	no	10	1	158	0	0	no	10	2	200–203	0.2	0.19	no
Ant5035	F-AGGATAGTTTCGCGGTTTATGGR-ACTGACTCGYAGTGTATTTGAGGT	10	2	340–342	0.4	0.33	no	10	9	412–442	0.3	0.94	yes	10	6	365–384	0.8	0.77	no	10	8	284–341	0.8	0.9	no	10	1	331	0	0	no	10	1	311	0	0	no
Ant7249	F-AAGTGTCAAGGGCGACTGAGR-CGGGGACAATGGAGCAATCA	10	1	425	0	0	no	10	7	320–359	0.4	0.86	yes	10	6	369–398	0.5	0.68	no	10	5	345–368	0.6	0.74	no	10	1	325	0	0	no	10	1	358	0	0	no
Ant7680	F-TCCCGGAGCAGCAATTATCCR-TAGGACAAAATGGAGCCCGC	10	1	306	0	0	no	9	11	332–386	0.56	0.97	yes	10	6	310–328	0.6	0.74	no	10	1	257	0	0	no	10	1	219	0	0	no	10	1	264	0	0	no
Ant8424	F-TCATAATGCAGATGATGGAACTCCTR-GGCGAGTAACACAATGGCAC	10	2	262–265	0.2	0.19	no	10	8	894–318	0.5	0.82	no	10	3	232–238	0.5	0.48	no	10	4	193–240	0.4	0.36	no	10	2	266–275	0.4	0.44	no	10	3	235–259	0.4	0.35	no
Ant8498	F-GATGCGAAGAGAGGCACGCGR-TGTTGCGAACYTAGGTGGCCTC	10	2	214–218	0.4	0.51	no	10	1	181	0	0	no	10	1	147	0	0	no	10	1	145	0	0	no	10	1	172	0	0	no	10	1	201	0	0	no
Ant9181	F-TGCCACTTACGCTGTGCACACR-AAATGCGGCCGAAGAGAAGA	10	1	280	0	0	no	no amplification						10	4	355–371	0.3	0.62	no	10	1	271	0	0	no	no amplification						no amplification					
Ant9218	F-GACCCACTTTGCCCTCGTAAR-CTCTCGATTAGTCAGGGTGGC	10	1	335	0	0	no	5	6	500–564	0.6	0.93	no	10	4	311–322	0.7	0.74	no	10	5	336–343	0.4	0.44	no	10	1	383	0	0	no	10	1	360	0	0	no
Ant10878	F-CGGGTGYTAGTCGTCGCCATR-GATCAATGCCGCAACGCTAA	10	1	302	0	0	no	10	7	358–377	0.6	0.88	no	10	3	292–298	0.6	0.51	no	10	8	280–321	0.8	0.86	no	10	2	283–285	0.1	0.1	no	10	1	320	0	0	no
Ant11315	F-AGCGTGTGCGACCGTGTAGCR-GCCATATATCATGGCTTGCCAG	10	1	358	0	0	no	10	1	380	0	0	no	10	1	317	0	0	no	10	1	355	0	0	no	10	1	322	0	0	no	10	1	343	0	0	no
Ant11400	F-CAACCACTTTGGGGCGCGAGR-CGAACCTCTTAATGAAATTCTCACCC	10	1	258	0	0	no	10	9	259–336	0.7	0.85	no	10	2	234–238	0.3	0.53	no	9	1	242	0	0	no	10	1	294	0	0	no	10	1	251	0	0	no
Ant11893	F-CAGGCTCGGRACGTTAATGCR-GGTGCCGACGTCTAGCTAGC	10	9	375–392	1	0.89	no	10	5	377–412	0.2	0.82	yes	10	4	390–398	0.5	0.73	no	10	1	336	0	0	no	10	1	321	0	0	no	10	4	343–358	0.6	0.66	no
Ant12220	F-AAAAGAGGCGGGCGTTCTTAR-GGTGTTCYGCCCCACCCGTA	10	1	378	0	0	no	no amplification						10	3	274–280	0.3	0.28	no	10	1	226	0	0	no	10	2	306–360	0.3	0.4	no	10	1	327	0	0	no

*n* is the number of individuals successfully genotyped for each locus, *A* is the number of alleles, *H_O_* is observed heterozygosity, *H_E_* is expected heterozygosity, and the last column for each species indicates whether that locus deviates from Hardy-Weinberg equilibrium in that species.

**Figure 2:**
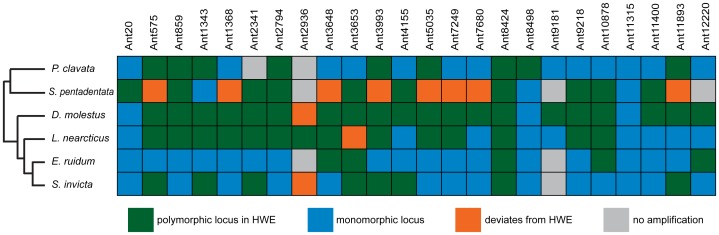
Overview results of genotyping 24 microsatellite loci for six different ant species. Green indicates loci that were polymorphic and in Hardy-Weinberg equilibrium, blue indicates monomorphic loci, orange indicates loci that were polymorphic but deviated from Hardy-Weinberg equilibrium, and grey indicates loci that did not amplify. The phylogeny to the left of the figure shows the evolutionary relationships of the species tested.

From those 45 loci, we selected 24 that amplified well in all or most of the six species tested and also had at least ten consecutive repeats of their motif in the genomes of more than one of the species with available genome sequences (Table S1 in File S1). We genotyped those 24 loci across all six species using fluorescently labeled primers (Applied Biosystems). PCR amplification was successful for all 24 loci in *L. nearcticus* and *D. molestus*, for 23 loci in *S. invicta*, for 22 loci in *P. clavata* and *E. ruidum*, and for 21 loci in *S. pentadentata* (Table 2, Figure 2). To determine which of the microsatellite loci were polymorphic in any given species, we genotyped ten individuals from ten different colonies from the same population of each species for each locus. On average, 12.83 (±6.15 SD) of the 24 loci were polymorphic in a given species, and 11.16 (±5.27 SD) were polymorphic and in Hardy-Weinberg equilibrium (Table 2, Figure 2). Across those polymorphic loci in Hardy-Weinberg equilibrium, the average number of alleles per locus per species was 4.59 (±2.41 SD). The average observed heterozygosity was 0.534 (±0.22 SD), and the average expected heterozygosity was 0.61 (±0.22 SD). Most of the loci were monomorphic for multiple species. However, in all cases the monomorphic allele at a given locus was different for each species. We found no statistical linkage disequilibrium (at p<0.00003 after Bonferroni correction) between any pair of loci in any species, but this is likely due to small sample sizes and reduced power due to the large number of tests performed. In fact, in all eight genomes there are scaffolds containing multiple loci, i.e. these loci occur on the same chromosome and are therefore physically linked (Table S2 in File S1).

## Discussion

To reduce the time and cost associated with developing microsatellite primers for a large number of different species, we designed a set of 45 primer pairs for potential use in a broad range of ant species spanning many millions of years of evolution. We tested 24 of these primer pairs in detail across six distantly related ant species from six different subfamilies. The number of useful polymorphic loci ranged from 5 to 20 for the six species we tested, although those loci were not always the same across species. Although we found no statistical linkage between any loci, some loci were located on the same scaffold in the genome assemblies of the reference species, and the location of the loci in the reference genomes should be considered when selecting primers from this set (Tables S1 & S2 in File S1). In assessing the utility of these markers in other species, it may be initially beneficial to test the entire set using inexpensive unlabeled primers. Then fluorescently labeled primers can be used for genotyping only those loci that amplify and yield clean PCR products. To further reduce costs, the primers described here could be used as unlabeled locus-specific primers in combination with universal labeled-tail primers [43].

Microsatellites have been an important tool for studies in population genetics for more than 20 years [1]–[3]. They are excellent markers for many types of studies including pedigree analyses and mating system studies, but their applicability has previously been limited by the narrow range of taxa in which each locus can be used. Researchers usually develop sets of primers specifically for their study species or a group of closely related species, and ants are no exception in this respect (e.g. [44]–[58]). For example, we found 32 publications of microsatellite primer notes for ants in the journal *Molecular Ecology Resources*, a leading outlet for the publication of population genetic markers. These primer notes represented 31 species and 28 genera. Looking only at those studies that described more than ten polymorphic loci per species, the number of alleles per locus ranged from 2 to 21 (Table 3). Species-specific primers often had more alleles per locus than we report here. The average number of alleles per locus across all species and loci from Table 3 is 7.58(±4.57 SD) while the average for the loci described here is 4.59 (±2.41 SD). One possible explanation is that this reflects a tradeoff between sequence variability within species and sequence conservation across species. On the other hand, this trend is probably at least partly attributable to our small sample size of specimens per species. The number of alleles per locus will likely increase as more samples are genotyped, especially if these come from different populations.

**Table 3:** Overview of number of alleles and expected and observed heterozygosity in eight studies of species-specific microsatellite primers in ants.

Species	Number of loci	Mean *A*	*A* range	Mean H_E_	H_E_ range	Mean H_O_	H_O_ range	Reference
*Allomerus octoarticulatus*	15	7.03	2–21	0.65	0.19–0.95	0.65	0.20–0.90	[51]
*Oecophylla smaragdina*	13	5.00	2–14	0.58	0.10–0.89	0.30	0.00–0.60	[52]
*Petalomyrmex phylax*	14	7.43	2–15	0.68	0.05–0.93	0.66	0.05–1.00	[53]
*Formica exsecta*	14	8.07	3–18	0.72	0.36–097	0.60	0.37–0.83	[54]
*Wasmannia auropunctata*	12	6.48	2–14	0.63	0.31–0.88	0.71	0.23–0.97	[55]
*Azteca ulei*	12	10.21	4–18	0.81	0.27–0.97	0.66	0.20–1.00	[56]
*Lasius austriacus*	11	9.91	4–19	0.78	0.19–0.93	0.70	0.20–0.90	[57]

Number of loci is the number of polymorphic loci described in that study, mean *A* is the average number of alleles per locus, *A* range is the range of allele numbers in each study, mean H_E_ and mean H_O_ are the average expected and observed heterozygosity respectively, and H_E_ range and H_O_ range are the ranges of expected and observed heterozygosity, respectively.

Many microsatellite primers are effective at amplification in congenerics, and some microsatellite primers have been successfully used across genera within the same ant subfamily e.g. [59–61]. However, to our knowledge, none have successfully amplified polymorphic microsatellite loci across multiple subfamilies. Here we characterize conserved microsatellite markers that are broadly useful across the ants and that will open opportunities for research on the many ant species lacking established genetic markers. These markers, like other microsatellites, will be especially useful for addressing questions in social insect research related to parentage, mating system and colony pedigree structure, i.e. questions for which it is preferable to maximize the number of samples genotyped while fewer markers are generally sufficient. The markers will also be useful in standard population genetic analyses, e.g. of population structure and gene flow. For questions that require a large number of markers such as genomic mapping, NGS data will generally be preferable. However, the loci presented here can readily be used to supplement NGS data.

There is demand for broadly applicable microsatellite primers outside the ants as well. Attempts to use microsatellite primers far outside of the species for which they were designed have had varying success. For example, primers designed for use in cattle have proven useful in other closely related mammals [19], [20], [62], and microsatellite primers designed for several different legumes have amplified polymorphic loci in the legume genus *Glycyrrhiza* [63]. Some primers designed for the paper wasp genus *Polistes* have also successfully amplified polymorphic loci in other Polistine wasps and even in the related subfamilies Vespinae and Stenogastrinae [18]. In marine turtles, primers have successfully amplified polymorphic microsatellites in species that diverged 300 MYA [23]. Additionally, a set of primers similar to those described here has been designed for birds using the genomes of the chicken, *Gallus gallus*, and the zebra finch, *Taenipygia guttata* [64], [65]. These conserved microsatellite loci also span a long evolutionary distance, as these species have diverged approximately 100 to 120 MYA [66], [67]. Our study in ants and those in birds [64], [65] present sets of primers designed explicitly for use in a broad range of species spanning a long evolutionary distance rather than testing species-specific primers in other distantly related species. Together, they set a precedent for identifying similar sets of markers in other diverse groups of comparable ages. This suggests that, with the availability of genomic information across an ever-increasing range of taxa, conserved microsatellites will become available as powerful population genetic tools for a wide variety of organisms.

## Materials and Methods

### Specimen collection

All specimens of *Ectatomma ruidum* and *Paraponera clavata* were collected at the Organization for Tropical Studies field station in La Selva, Costa Rica. *Simopelta pentadentata* specimens were collected in Monteverde, Costa Rica. *Dorylus molestus* specimens were collected in Kakamega Forest, Kenya. *Lasius nearcticus* specimens were collected at the Rockefeller University Center for Field Research in Millbrook, New York, USA, and specimens of *Solenopsis invicta* were collected in Tallahassee, Florida, USA.

Collection permits were acquired for all samples where necessary. A permit for specimens from Kakamega National Park, Kenya was granted by the National Council for Science and Technology (permit number NCST/RCD/12B/012/37B). A permit for specimens from Costa Rica was granted by Ministerio de Ambiente, Energia y Telecomunicaciones (permit number 192-2012-SINAC). Permits were not required for specimens collected in the United States. No protected species were sampled.

### Bioinformatics

Seven available ant genomes were downloaded from Ant Genomes Portal (hymenopteragenome.org/ant_genome), and our lab has recently published the *C. biroi* genome [42]. The genome versions for each species were *A. cephalotes* v1.0, *A. echinatior* v2.0, *C. floridanus* v3.3, *C. biroi* v2.0, *H. saltator* v3.3, *L. humile* v1.0, *P. barbatus* v3.0, *S. invicta* v1.0. Microsatellites in the *C. biroi* genome were located using Tandem Repeats Finder ('TRF'; v. 4.04) [68], which utilizes Smith-Waterman style local alignment. Tandem repeats are reported only if they exceed a minimum alignment score, specified as 50 (Minscore  = 50). Alignment mismatches were assigned a weight of five (Mismatch  = 5). Additionally, the size of the repeat pattern was limited to five bases (Maxperiod  = 5). The microsatellite indices returned were used to generate a masked BLAST query for each microsatellite, extended to include 200-bp flanking regions. The query sequence was used to search all eight sequenced ant genomes, including *C. biroi*, using BLAST (v. 2.2.26+) [69]. The results were filtered to remove matches with less than 60% identity. Microsatellite flanking regions that generated unique BLAST hits in all eight genomes were aligned using MUSCLE [70]. To confirm that these conserved flanking regions indeed contained microsatellite sequences, TRF was used to search for microsatellites in all database genomes at the indices returned by BLAST for each hit (settings as stated above). Primer3 software (v. 2.3.4; http:/primer3.sourceforge.net/releases.php) [71] generated primers from the consensus sequence in each flanking region. A maximum of four unknown bases were allowed in any primer set (PRIMER_MAX_NS_ACCEPTED  = 4). All unspecified parameters used the default or recommended settings. Custom Python scripts were used to parse TRF and Primer3 outputs, prepare files for BLAST and Primer3, and filter the BLAST results. These scripts are available upon request from the corresponding author. Initially, 176 loci were identified across all genomes with the described bioinformatics pipeline, from which we chose 45 loci for further study. These 45 loci were chosen subjectively based on the number of perfect repeats in different species and the presence of a microsatellite motif in as many ant genomes as possible.

### DNA extraction, PCR amplification and genotyping

DNA was extracted by first homogenizing the tissue in a Qiagen TissueLyser II and then heating the sample at 96°C for 15 minutes in 200 µl of 10% Chelex in TE solution. The samples were then centrifuged at 9100 rpm for three minutes, and the supernatant containing the DNA was removed and used as the template for PCR amplification.

The PCR cocktail (10 µl total volume) for all reactions contained 1 µl PCR Gold Buffer (10x), 0.5 µl MgCl_2_ (25 mM), 0.5 µl dNTPs (10 mM total, 2.5 mM each), 0.1 µl of each forward and reverse primer (10 µM), 0.1 µl AmpliTaq Gold (5 U/µl), 1 µl DNA template and 6.7 µl H_2_O. PCR reactions were run on an Eppendorf Mastercycler Pro S under the following conditions: 10 min at 95°C followed by 40 cycles of 15 s at 94°C, 30 s at 55°C and 30 s at 72°C, and a final extension of 10 min at 72°C. PCR products were sent to a commercial facility (Genewiz, Inc.) for genotyping. Analysis of chromatograms was performed using PeakScanner (Applied Biosystems). Calculations of observed and expected heterozygosity, as well as tests for linkage disequilibrium and deviations from Hardy-Weinberg equilibrium were performed using F-STAT (v2.9.3.2) [72].

## Supporting Information

File S1 Contains Tables S1 and S2 described below. **Table S1.** Details of microsatellite loci in eight ant genomes. Numbers in parentheses behind the size of the targeted fragment indicate that there are a number of unknown ("N") bases inserted into the available genome sequence. In some cases, these can be larger stretches of "N" bases in the published genome assembly. This number is included in the total size of the targeted fragment. In most cases, this implies that the given size of the targeted fragment is probably imprecise. The column "Size of targeted fragment in base pairs" thus gives "Total base pairs (number of N bases among the total base pairs)". Some loci have multiple motifs listed. All motifs are in the same region and are included in the size of the targeted fragment. **Table S2.** Physical linkage of microsatellite loci. X indicates where two loci are on the same scaffold in that species. Order of species left to right in every box is *P. barbatus, H. saltator, At. cephalotes, Ce. biroi, L. humile, Ca. floridanus, S. invicta, Ac. echinatior.*
(XLSX)
